# The interplay between neuroinflammation and endothelial dysfunction in cerebral small vessel disease

**DOI:** 10.3389/fimmu.2026.1809556

**Published:** 2026-05-21

**Authors:** Cong Jin, Yang Zhao

**Affiliations:** Department of Neurology, The First Hospital of Jilin University, Changchun, Jilin, China

**Keywords:** blood-brain barrier, cerebral small vessel disease, endothelial dysfunction, neuroinflammation, neurovascular unit

## Abstract

Cerebral small vessel disease (CSVD) is a leading cause of ischemic stroke and vascular cognitive impairment. Despite well-defined neuroimaging features and a substantial clinical burden, the underlying pathological mechanisms of CSVD remain poorly understood, and effective interventions are still lacking. Current human and experimental evidence suggest that endothelial dysfunction and neuroinflammation are closely coupled and may form a mutually reinforcing process in many CSVD-relevant settings. This review synthesizes available evidence into a working framework in which vascular risk factors promote endothelial phenotypic switching and blood-brain barrier (BBB) dysfunction, thereby facilitating neuroinflammatory activation. Pericytes, astrocytes, and microglia within the neurovascular unit can then amplify barrier injury and inflammatory signaling through context-dependent feedback interactions, including matrix metalloproteinase-mediated proteolysis. In turn, the chronic inflammatory milieu may sustain persistent endothelial dysfunction or alter endothelial responsiveness to subsequent inflammatory stimuli, potentially allowing pathological processes to continue even after the original triggers are attenuated. Based on this evidence-guided framework, we also evaluate therapeutic strategies directed at distinct nodes of the endothelial dysfunction-neuroinflammation network.

## 1 Introduction

Cerebral small vessel disease (CSVD) is a heterogeneous disorder affecting the brain’s small perforating arteries, arterioles, capillaries, and venules, and it is a major cause of stroke, vascular cognitive impairment, and dementia in older adults (1–3). Its clinical spectrum ranges from incidental neuroimaging abnormalities to progressive gait, mood, and cognitive disorders, while its radiological hallmarks include white matter hyperintensities, lacunar infarcts, cerebral microbleeds, and enlarged perivascular spaces (1). With population ageing, the growing prevalence of CSVD has become an important clinical and societal challenge.

Although CSVD is defined clinically and radiologically, its pathogenesis is not uniform. Different subtypes likely arise from partially distinct initiating mechanisms. Even so, patient-based and experimental studies indicate that endothelial dysfunction, blood-brain barrier (BBB) injury, and inflammatory signaling interact closely across many CSVD-relevant settings (4–8). In patients, endothelial biomarker abnormalities correlate with CSVD burden, and BBB leakage has been demonstrated in lacunar stroke and white matter hyperintensities (5, 8, 9). Experimental studies further show that correcting endothelial dysfunction can attenuate white matter vulnerability (4). Together, these findings support endothelial dysfunction as a recurrent pathogenic axis and a plausible downstream point of convergence in CSVD, rather than proof of one universal initiating sequence.

In this review, endothelial injury refers to the initial structural or molecular insult to endothelial cells. Endothelial phenotypic switching refers to the transition from a quiescent state to an activated phenotype that is pro-inflammatory, pro-thrombotic, and barrier-disruptive. Endothelial dysfunction refers to the impairment or loss of normal endothelial homeostatic functions (10). We focus primarily on atherosclerotic CSVD. We first outline the core mechanism most directly supported by current evidence: endothelial injury and phenotypic switching, BBB disruption, neurovascular-unit inflammatory amplification, and persistent endothelial dysfunction within a chronic inflammatory milieu. We then discuss potential modifiers of this framework, including vessel-segment-specific vulnerability and memory-like endothelial state transitions. At present, these modifiers should be viewed as emerging rather than established components of the core mechanism (11–22).

### 1.1 Endothelial homeostasis and dysfunction in CSVD pathogenesis

#### 1.1.1 Primary endothelial homeostasis and its disruption in CSVD: from physiological functions to pathological phenotype

CSVD encompasses several pathological subtypes, and this review focuses primarily on atherosclerotic CSVD (2, 23). Within this context, the cerebral microvascular endothelium should be regarded not merely as a passive target of injury, but as a key regulatory hub for vascular homeostasis and a potential early pathogenic interface at which multiple vascular risk factors may converge to promote disease evolution (4, 5, 8).

Under physiological conditions, cerebral microvascular endothelial cells maintain vascular homeostasis by regulating vascular tone, preserving an anti-thrombotic luminal surface, limiting leukocyte adhesion and transmigration, and supporting vascular repair (24, 25). In the brain, these functions are coupled to BBB-specialized endothelial programs documented in primary profiling and vascular-mapping studies, including tight-junction enrichment, transporter expression, and zonation-dependent endothelial specialization along the cerebrovascular tree (11–15). These roles are summarized here as foundational endothelial biology that provides the physiological baseline for interpreting CSVD-associated phenotypic change, rather than as mechanisms proven uniquely in CSVD.

These homeostatic programs are vulnerable to diverse yet converging insults. Chronic hypertension reduces endothelial transforming growth factor-beta-activated kinase 1 (TAK1) expression, disinhibits receptor-interacting serine/threonine-protein kinase 1 (RIPK1) and mixed lineage kinase domain-like protein (MLKL) signaling, and promotes endothelial necroptosis accompanied by tight-junction degradation (26). Hyperglycaemia can also activate oxidative and inflammatory pathways in endothelial systems; primary studies show high-glucose-driven NLR family pyrin domain containing 3 (NLRP3) and nuclear factor kappa B (NF-κB)-associated endothelial inflammatory activation *in vitro* and in diabetic vascular models (27–29). Hypercholesterolaemia induces nicotinamide adenine dinucleotide phosphate (NADPH) oxidase-derived oxidative stress, upregulates P-selectin, and promotes a pro-adhesive endothelial phenotype (30). Aging is likewise associated with reduced nitric oxide bioavailability and with CSVD-relevant methylarginine abnormalities, while endothelial nitric oxide synthase (eNOS) deficiency aggravates white matter injury in a spontaneous chronic hypoperfusion model (31–33). Among these initiating pathways, the TAK1-RIPK1-MLKL axis is supported by a CSVD-specific primary study (26), whereas the hyperglycaemia- and ageing-related mechanisms are drawn mainly from broader endothelial and CSVD-relevant primary literature rather than from one single CSVD-specific model. Although these initiating factors differ in origin and signaling profile, they converge on a shared outcome: the disruption of endothelial homeostasis and the emergence of pathological endothelial phenotypes that set the stage for downstream BBB failure and neurovascular inflammation.

#### 1.1.2 BBB-relevant endothelial specialization across the small-vessel tree

The small-vessel tree refers to the network of cerebral microvessels, including arterioles, capillaries, and venules. Primary single-cell and vascular-mapping studies show that the cerebrovascular tree is zonated rather than uniform. Along the arteriovenous axis, endothelial transcriptional programs change systematically. Mural-cell organization also shifts from concentric smooth-muscle coverage in arteries and arterioles to ensheathing and then capillary pericytes as vessel caliber narrows (11–13). Capillary endothelial cells show the most canonical BBB phenotype. They suppress transcytosis via major facilitator superfamily domain-containing protein 2A (MFSD2A) and show prominent aging-related transcriptomic changes linked to barrier and metabolic dysfunction (14, 15). Recent single-cell studies also identified specialized post-capillary venular endothelial populations. These populations are enriched for adhesion molecules and leukocyte transmigration functions, suggesting that immune-cell trafficking is not evenly distributed along the vascular tree (34).

These primary findings provide biological context for differential vulnerability across vascular segments. *In vitro* studies show that nonphysiological pulsatility and high shear directly impair brain-endothelial barrier features (17). Animal studies also show that pericyte loss produces zonation-dependent endothelial changes, including reduced BBB-specialized programs and increased permeability-related signatures (18). Together, these observations support endothelial heterogeneity as relevant background biology for CSVD. However, they do not yet explain why human CSVD localizes to particular small-vessel segments. Thus, this inference remains as an emerging concept rather than a core established mechanism.

These segment-specific endothelial programs may also help explain why pathology is preferentially expressed within the small-vessel tree. Small perforating arteries and arterioles face marked pulsatility and autoregulatory stress. Chronic vascular risk factors may therefore disrupt endothelial homeostasis early at these sites. Capillary endothelial cells carry the most specialized BBB phenotype and depend strongly on pericyte-supported barrier programs. This may make them especially vulnerable to aging, abnormal flow, and pericyte loss. Post-capillary venules provide a distinct inflammatory interface. Endothelial populations in this segment are enriched for leukocyte adhesion and transmigration programs.

Collectively, these features suggest that CSVD is not a uniform endothelial disorder across all vessel types. Instead, it is better viewed as a small-vessel network pathology in which segment-specific vulnerabilities promote hemodynamic stress, barrier dysfunction, and inflammatory amplification along the perforating arteriolar-capillary-venular axis. This interpretation remains biologically plausible, but studies have not yet demonstrated the full sequence in human sporadic CSVD. Thus, endothelial heterogeneity along the small-vessel tree shapes the regional vulnerability pattern of CSVD. Taken together, these converging insults compromise endothelial homeostatic functions across vulnerable vascular segments. Segment-specific endothelial programs set the stage for a more destructive transition: from endothelial dysfunction to persistent neuroinflammatory amplification.

The next section examines how eNOS uncoupling acts as a critical molecular switch linking early risk-factor exposure to BBB disruption and subsequent inflammatory cascades within the neurovascular unit (NVU).

### 1.2 Initiation of endothelial dysfunction: from eNOS uncoupling to a pro-inflammatory phenotype

In CSVD, risk factors can disturb endothelial equilibrium and shift endothelial cells from a protective, homeostatic phenotype toward an activated, barrier-disruptive state. In patients, reduced endothelial function and abnormal endothelial biomarkers are associated with CSVD burden (5, 8), while experimental reversal of endothelial dysfunction lessens white matter vulnerability (4). These observations support endothelial dysfunction as a common downstream pathological pathway in CSVD, although they do not establish one universal initiating event across all subtypes (3).

Oxidative stress, triggered by vascular insults such as oxidized low-density lipoprotein (oxLDL) and hyperhomocysteinemia-associated endothelial injury, plays a central role (35–37). Under pathological conditions, it depletes the essential eNOS cofactor tetrahydrobiopterin (BH_4_). Superoxide anion (O_2_^-^) reacts with NO to form peroxynitrite (ONOO^-^), which oxidizes BH_4_ to its inactive form. BH_4_ deficiency leads to eNOS uncoupling, whereby electron flow is diverted from NO synthesis to molecular oxygen, generating more O_2_^-^. This establishes a self-amplifying cycle (BH_4_ oxidation → eNOS uncoupling → O_2_^-^ production → NO quenching/ONOO^-^ formation → further BH_4_ oxidation), perpetuating oxidative stress and endothelial dysfunction (38). This process can impair endothelium-dependent vasodilation and provides a plausible endothelial basis for subsequent neurovascular-coupling and cerebral blood-flow autoregulatory dysfunction (39).

More quantitative evidence comes from oxidative stress-related animal models and complementary human imaging studies relevant to sporadic CSVD. In angiotensin II-related models, neurovascular dysfunction develops early. Acute ANG II impairs neurovascular coupling through NADPH oxidase-derived radicals (40), and in a 14-day ANG II paradigm the whisker-evoked CBF response fell from 18.5 ± 0.8% to 14.2 ± 0.6%; a similar reduction was seen after 7 days of IL-17A infusion, from 20.0 ± 1.1% to 14.1 ± 1.1% (41). Over a longer time course, 4 weeks of chronic ANG II hypertension altered cerebrovascular autoregulation, with aged hypertensive mice showing loss of autoregulatory protection (42, 43). Complementary evidence further suggests that persistent flow-regulatory dysfunction is followed by measurable white-matter injury. In BCAS mice, parenchymal CBF fell to approximately 49-55% of baseline on day 1, remained near this level through day 14, and recovered only partially by day 28 (44). In a separate BCAS study, the median white-matter injury score in the medial corpus callosum reached 1.0 (IQR 0.75-1.0) by postoperative day 30, compared with 0.25 (IQR 0-0.5) in sham controls (45). Consistent with these experimental findings, a longitudinal human MRI study showed that regions of normal-appearing white matter that later converted to WMH had 26.5% lower baseline cerebrovascular reactivity than contralateral homologous white matter, and after 1 year the resulting lesions showed 8.7% higher T2 and 17.0% higher mean diffusivity (46). A recent population−based cohort study extends that sustained declines in CBF and cerebrovascular reactivity independently predict WMH progression, with risk ratios reaching 1.36 (95% CI: 1.19−1.55) for the lowest CBF quartile (47). Together, these findings suggest that sustained neurovascular and flow-regulatory dysfunction is followed by subsequent white-matter injury over periods ranging from weeks in mice to one year in humans. Moreover, multiple lines of evidence—oxidative-flux modeling, phase−specific autophagic responses, and human hemodynamic cohorts—point to the same conclusion. The injury process is not a single discrete event but a protracted, stage−dependent pathological cascade.

### 1.3 Disruption of the blood-brain barrier: from structural compromise to neuroinflammatory triggering

The pro-inflammatory shift in endothelial phenotype directly compromises BBB function. In spontaneously hypertensive stroke-prone rats (SHRSP), BBB disruption is accompanied by reduced levels of the tight junction protein claudin-5 (48, 49). In parallel, an *in vitro* flow study using human brain microvascular endothelial cells showed that physiological shear enhanced tight-junction marker expression, whereas high shear stress and/or pulsatility reduced junction-related proteins and altered junctional morphology (17).

Once the blood-brain barrier is disrupted, plasma components leak into the brain parenchyma. Patients with lacunar stroke and white matter hyperintensities show this leakage *in vivo* (6, 7). Data linking blood-derived proteins to neuroinflammation are more model dependent. Mouse studies support the idea that fibrinogen acts as a danger signal. In one *in vivo* experiment, researchers delivered wild-type plasma stereotactically into the brain and then analyzed sorted microglia by RNA sequencing. Fibrinogen deficiency markedly reduced the induced microglial transcriptional response (50). Evidence for the downstream matrix metalloproteinase (MMP) cascade comes mainly from hypoxia and ischemia models. Studies of hypoxia biology and experimental ischemia support hypoxia-inducible factor-1α (HIF-1α)-furin-MT1-MMP/MMP-2 signaling (51–53). Rat reperfusion-injury and glial-culture studies suggest that astrocytes are a major source of MMP-2 and that microglia/macrophages contribute MMP-9 (54, 55). Rat focal ischemia models also show direct cleavage of tight-junction proteins (56). In SHRSP white-matter injury models, anti-inflammatory or MMP-modulating treatment attenuated hypoxia-induced injury (57). These findings support the pathological relevance of this proteolytic axis. In human CSVD, the safest conclusion is that BBB leakage likely amplifies neuroinflammation and protease-mediated barrier injury. Much of the detailed molecular sequence still comes from animal and *in vitro* studies (3, 57–59).

Taken together, the evidence supports a working model in which BBB leakage and hypoperfusion jointly drive a fibrinogen-microglia inflammatory trigger and a HIF-1α-MMP proteolytic cascade. This interaction creates a self-reinforcing cycle of barrier injury. Human CSVD evidence is strongest for BBB leakage itself (6, 7). The fibrinogen-microglia and protease sequence still rely mainly on animal, ischemia/hypoxia, and *in vitro* studies (50–57). For that reason, this framework is best interpreted as a convergent, model-integrated mechanism rather than a single continuous molecular sequence already demonstrated in human CSVD tissue. Even so, it provides a useful starting point for translational studies, particularly those using single-cell and spatially resolved analyses of human white matter lesions.

A segment-specific view may refine this model. Capillary endothelial cells carry the most specialized BBB program, so barrier failure in CSVD may be especially relevant at the capillary level. Upstream arteriolar dysfunction and downstream venular responses likely shape when and where this failure becomes clinically significant.

These considerations help localize where barrier failure may be most consequential within the small-vessel tree, but they do not by themselves explain how endothelial injury is converted into sustained tissue-level inflammation. The next section therefore shifts from segmental vascular vulnerability to multicellular amplification, focusing on how pericytes, astrocytes, and microglia transform barrier disruption into a persistent neuroinflammatory response.

### 1.4 Amplification of neuroinflammation: cellular network synergy and positive feedback

This cascade reveals how cells within the neurovascular unit engage in complex positive-feedback interactions that amplify initial injury signals into a sustained inflammatory response.

#### 1.4.1 Endothelial-pericyte axis dysfunction: pericyte loss, activation, and barrier instability

Following endothelial phenotypic switching and early BBB impairment, pathological changes in CSVD are unlikely to remain confined to endothelial cells alone. Because barrier integrity depends on close endothelial-pericyte coupling, disruption of endothelial homeostatic signaling may secondarily impair pericyte recruitment, vascular coverage, and support functions (18, 60–62). In particular, endothelial PDGF-BB/PDGFRβ signaling is a key pathway for pericyte recruitment and vascular stabilization (60), whereas excessive vascular endothelial growth factor (VEGF) signaling may interfere with pericyte recruitment and maturation (63, 64). Vasoactive imbalance, including reduced nitric oxide (NO) bioavailability, may further weaken endothelial-pericyte communication (4, 61, 62). Experimental studies also show that loss of pericyte contact is accompanied by endothelial barrier abnormalities, including increased plasmalemma vesicle-associated protein (PLVAP) expression, reduced major facilitator superfamily domain-containing 2A (MFSD2A) expression, and increased permeability (18). Within this framework, dysfunction of the endothelial-pericyte axis is better viewed not as an isolated initiating event, but as a potential amplification step through which early endothelial injury is converted into more persistent barrier instability.

Current support for this interpretation, however, comes mainly from animal and *in vitro* studies rather than from direct studies of sporadic human CSVD. In experimental chronic cerebral hypoperfusion, reduced pericyte coverage in the corpus callosum is accompanied by increased BBB permeability (65). By contrast, human evidence remains largely correlational. Postmortem studies in age-related dementias, including vascular dementia and post-stroke dementia, show reduced deep white matter pericyte numbers in association with BBB disruption (66). However, such observations do not establish temporal sequence or causality in sporadic CSVD. Thus, a cautious interpretation is that pericyte dysfunction may represent one component of the pathogenic cascade in CSVD, although its precise position and importance in human disease remain to be clarified (65–68).

Pericyte-derived MMP-9 has been suggested as a possible contributor to barrier destabilization, but current evidence comes mainly from *in vitro* and acute injury models; its relevance to chronic human CSVD remains to be clarified (69–71).

Overall, existing studies from animal models, *in vitro* experiments, postmortem correlational observations, and related biomarker-oriented work suggest that pericyte abnormalities may participate in the pathophysiology of human CSVD, but their precise mode of action, temporal sequence, and causal role remain unresolved (65, 66, 72–74). Taken together, these findings suggest that pericyte abnormalities may destabilize the barrier and reshape the perivascular inflammatory milieu, even though their exact causal role in sporadic human CSVD remains uncertain. Once the endothelial-pericyte axis is disturbed, the pathological signal is no longer confined to the vessel wall. It can spread through glial communication networks, particularly the reciprocal dialogue between astrocytes and microglia.

#### 1.4.2 Astrocyte-microglia dialogue: positive feedback amplification of inflammatory signals

Under conditions of endothelial dysfunction and chronic hypoperfusion, astrocytes adopt a pro-inflammatory phenotype. This shift makes them a critical amplification node within the endothelial injury-neuroinflammation vicious cycle. Reactive astrocytes are not passive responders. Instead, they convert barrier leakage and hypoxia-associated cues into chemokine signals, chiefly C-C motif chemokine ligand 2 (CCL2) and C-X3-C motif chemokine ligand 1 (CX3CL1) (75–77). These mediators recruit and prime nearby microglia (75–77). Activated microglia then release interleukin-1 beta (IL-1β) and TNF-α. In turn, these cytokines intensify astrocytic reactivity and transform a transient stress response into a positive-feedback loop (75–77). In this setting, microglia acquire a predominantly M1-like pro-inflammatory phenotype, with increased inducible nitric oxide synthase (iNOS) and IL-1β/TNF-α production. Astrocytes concurrently adopt A1-like features, marked by complement component C3 expression and loss of homeostatic support functions (75–77).

Evidence from prolonged cerebral hypoperfusion models, particularly bilateral common carotid artery stenosis (BCAS) mice combined with chronic hypoxia-conditioned astrocyte cultures, shows that expansion of A1-like astrocytes parallels white matter injury and impaired oligodendrocyte maturation (78). This pattern suggests that glial polarization is not merely an epiphenomenon. Rather, it actively amplifies tissue damage. By increasing the release of cytokines and reactive oxygen/nitrogen species, these polarization states enhance endothelial activation and further compromise blood-brain barrier integrity.

Beyond chemokines, purinergic P2X purinergic receptor 7 (P2X7) receptor-driven inflammasome signaling provides a second layer of amplification. ATP (adenosine triphosphate) released from injured cells and reactive astrocytes engages microglial P2X7 receptors. This interaction promotes NLRP3 inflammasome assembly and sustained IL-1β maturation. Much of the P2X7-NLRP3 mechanism has been characterized in experimental ischemia models, and its relevance to CSVD is supported by spontaneously hypertensive rat models. In these models, pharmacological NLRP3 inhibition with MCC950 attenuated microglial and astrocytic activation, blood-brain barrier breakdown, white matter injury, and endothelial dysfunction (79). These findings suggest that, once triggered, inflammasome-dependent signaling can maintain a self-sustaining inflammatory state even when the original endothelial injury is no longer dominant.

Complement-dependent crosstalk adds another feed-forward loop. In rats with chronic cerebral hypoperfusion induced by bilateral common carotid artery occlusion, aberrant microglial activation aggravates white matter injury through the complement component 3–complement component 3a receptor (C3-C3aR) pathway (80). Within the broader astrocyte-microglia dialogue, A1-like astrocytes are an important source of C3. This complement protein opsonizes myelin and cellular debris. It also pushes microglia toward a phagocytic activation state and links debris clearance to further TNF-α and IL-1β release (77, 80). Chronic cerebral hypoperfusion models, including BCAS mice and rats with bilateral common carotid artery occlusion, also show a temporal shift in glial responses. Microglial activation appears early, whereas astrocytic reactivity becomes more prominent as white matter injury evolves (78, 80). As endothelial activation and blood-brain barrier dysfunction progress, this glial crosstalk can also promote leukocyte trafficking. The extent of that infiltration likely varies by disease stage and vascular segment rather than occurring uniformly throughout CSVD (16, 78, 80). Vascular-mapping data support this view. They show that post-capillary venular endothelial populations are preferentially equipped for leukocyte adhesion and transmigration, so inflammatory cell entry is unlikely to be distributed evenly across the small-vessel tree. At later stages, astrocytes and microglia become functionally interdependent (78, 80).

The astrocyte-microglia dialogue therefore does not operate in isolation. It acts as an amplification system. This system converts transient endothelial injury into a durable, self-amplifying inflammatory signal that feeds back onto the endothelium, sustains endothelial dysfunction, and locks the neurovascular unit into a vicious cycle. Much of the evidence for these detailed molecular loops comes from BCAS mice, rats with bilateral common carotid artery occlusion, focal ischemia or reperfusion-injury models, and glial or hypoxia-conditioned cell-culture systems, rather than from direct analyses of human CSVD tissue. In human CSVD, the evidence for chronic glial activation is much stronger than that for the full intercellular signaling architecture. Within those evidentiary limits, this crosstalk remains a plausible route by which transient endothelial injury is converted into persistent neurovascular-unit inflammation that feeds back onto endothelial dysfunction.

This cytokine- and complement-driven crosstalk is only one arm of glial amplification. A second arm is proteolytic. The next subsection examines how astrocyte-associated protease activity may convert inflammatory signaling into structural blood-brain barrier damage.

#### 1.4.3 Astrocyte-mediated proteolysis: exacerbating BBB leakage and inflammation

Activated astrocytes may also contribute to proteolytic barrier injury, although the evidence is more fragmentary than for astrocyte-microglia cytokine crosstalk. Astrocytic MMP expression has been documented in glial-culture studies (55), and MMP-mediated cleavage of tight-junction proteins has been demonstrated in experimental focal ischemia (56). In SHRSP white matter injury, minocycline-sensitive neuroinflammatory damage further supports the pathological relevance of protease-associated barrier injury (57). Together with broader concepts of extracellular-matrix inflammation in vascular cognitive impairment (81), these data suggest that astrocyte-associated proteolysis may exacerbate BBB leakage and white matter injury; however, the exact astrocyte-specific contribution has not been directly resolved in human CSVD.

These interacting pathways form an efficient signal-amplification network. Microglia recruited through the endothelial-pericyte axis can feed into astrocyte-microglia signaling loops. Astrocyte-associated proteolysis may further weaken the BBB and increase parenchymal exposure to plasma-derived inflammatory factors. However, most detailed steps in this scheme derive from chronic hypoperfusion, ischemia, or cell-culture models rather than direct human CSVD tissue analysis (54–57, 69–71, 78–81). At present, the strongest conclusion is therefore that neurovascular-unit cells likely cooperate to prolong neuroinflammation after endothelial injury, even though the precise sequence and cell-specific weighting remain model-dependent.

**Figure 1:**
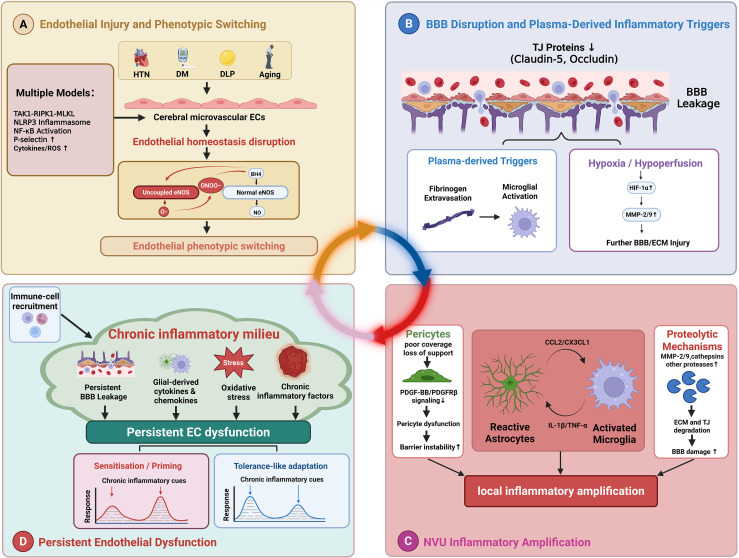
Endothelial injury–neuroinflammation network in cerebral small vessel disease. This figure summarizes the main pathological steps linking endothelial injury, blood–brain barrier disruption, neurovascular-unit inflammatory amplification, and persistent endothelial dysfunction in cerebral small vessel disease. **(A)** Endothelial injury and phenotypic switching. Vascular risk factors, including hypertension, diabetes mellitus, dyslipidaemia, and ageing, disturb cerebral microvascular endothelial-cell homeostasis. Oxidative stress may promote tetrahydrobiopterin depletion and endothelial nitric oxide synthase uncoupling, leading to reduced nitric oxide bioavailability and increased formation of superoxide anion and peroxynitrite. These changes favor endothelial injury and a shift toward a pro-inflammatory, pro-thrombotic, and barrier-disruptive phenotype. Additional model-supported pathways, including transforming growth factor-beta-activated kinase 1–receptor-interacting serine/threonine-protein kinase 1–mixed lineage kinase domain-like protein signaling, NLR family pyrin domain containing 3 inflammasome activation, nuclear factor kappa B activation, P-selectin upregulation, and cytokine/reactive oxygen species production, are shown as related mechanisms. **(B)** Blood–brain barrier disruption and plasma-derived inflammatory triggers. Endothelial dysfunction and hemodynamic stress weaken tight-junction integrity, including claudin-5 and occludin, and promote blood–brain barrier leakage. Plasma-derived proteins such as fibrinogen may then contribute to microglial activation. Hypoxia or hypoperfusion may also engage hypoxia-inducible factor-1 alpha-associated matrix metalloproteinase signaling and further aggravate blood–brain barrier and extracellular-matrix injury. **(C)** Neurovascular-unit inflammatory amplification. Within the neurovascular unit, pericyte-associated barrier instability, astrocyte–microglia crosstalk, and proteolytic mechanisms can amplify local inflammation. Reduced platelet-derived growth factor-BB/platelet-derived growth factor receptor beta signaling is shown as one mechanism linking endothelial–pericyte dysfunction to barrier instability. Astrocyte-derived C-C motif chemokine ligand 2 and C-X3-C motif chemokine ligand 1, together with microglial interleukin-1 beta and tumour necrosis factor alpha, form a reciprocal inflammatory loop. Matrix metalloproteinases and related proteases may further damage extracellular-matrix and tight-junction components. **(D)** Persistent endothelial dysfunction in a chronic inflammatory milieu. Persistent blood–brain barrier leakage, glial cytokines and chemokines, oxidative stress, and chronic inflammatory cues may sustain endothelial dysfunction. Repeated or prolonged inflammatory exposure may also diversify endothelial responses toward sensitization/priming or tolerance-like adaptation. These patterns should be viewed as heterogeneous endothelial-cell response states within persistent endothelial dysfunction, not as evidence of complete recovery. Context-dependent immune-cell recruitment may further reinforce this inflammatory milieu. Dashed elements indicate emerging, indirect, or model-dependent evidence. Created with BioRender.com.

Together, these interacting pathways suggest that neuroinflammatory amplification in CSVD is not a single linear cascade, but a coordinated network of cytokine, complement, and protease signals. Once this network becomes sustained, its effects extend beyond ongoing tissue inflammation and feed back onto endothelial cells themselves. The following subsection therefore considers how a chronic inflammatory milieu may maintain persistent endothelial dysfunction and diversify endothelial response states over time. To facilitate reading, **Figure 1** provides a schematic illustration of the endothelial injury-neuroinflammation interactions discussed above.

### 1.5 Persistent endothelial dysfunction and heterogeneous endothelial states in the inflammatory microenvironment

Once neuroinflammatory amplification is established, endothelial injury does not simply resolve in a linear manner. Instead, BBB disruption, blood-derived inflammatory signals, glial activation, oxidative stress, hypoperfusion, and hypoxia-related signaling continue to interact within the NVU, sustaining a chronic inflammatory milieu. Human CSVD data support this persistence at the systems level. Higher baseline BBB leakage predicts subsequent cognitive decline *in vivo*. BBB permeability and microglial activation may coexist without complete spatial overlap. Moreover, plasma from patients with early CSVD can activate inflammatory pathways in brain endothelial cells (6, 7, 82–84). In this context, persistent endothelial dysfunction refers to a prolonged loss of endothelial homeostatic functions under continuing inflammatory stress. It should not be interpreted as proof of one uniform, autonomously maintained endothelial inflammatory state. Rather, it describes an ongoing imbalance. In this imbalance, worsening endothelial dysfunction further destabilizes barrier integrity, sustains inflammatory signaling, and continues to support neurovascular injury (4, 85–87).

Within this chronic inflammatory milieu, endothelial cells may also undergo dynamic and context-dependent reprogramming. Emerging evidence suggests that repeated or prolonged inflammatory stimulation can induce memory-like changes. As a result, subsequent endothelial responses shift toward sensitization/priming or toward tolerance-like adaptation, depending on the nature, intensity, duration, and sequence of prior stimuli (19–22, 88, 89). Current support for this concept comes mainly from endothelial stimulation systems rather than direct studies of CSVD tissue. Repeated inflammatory exposure can produce durable changes in endothelial transcriptional programs and chromatin accessibility. These changes have downstream effects on endothelial-immune interactions, including enhanced monocyte adhesion after restimulation (19). Toll-like receptor-related studies further show that endothelial adaptation is bidirectional rather than uniform. For example, polyinosinic: polycytidylic acid (Poly I:C) priming can potentiate later inflammatory responses. By contrast, lipopolysaccharide (LPS), Pam3Csk4, or monophosphoryl lipid A priming can attenuate subsequent LPS-driven activation in a tolerance-like manner (20, 21). In addition, oxLDL has been shown to induce pro-inflammatory memory-like reprogramming in endothelial cells. This reprogramming is accompanied by metabolic and epigenetic alterations, increased adhesion-molecule expression, and enhanced leukocyte-endothelial interactions (22). These findings suggest that chronic inflammatory exposure may diversify endothelial behavior over time. However, direct evidence for such heterogeneous endothelial states in sporadic human CSVD remains limited.

Importantly, tolerance-like adaptation should not be equated with recovery of endothelial homeostasis. A weaker response to a secondary stimulus does not mean that BBB integrity, vasoregulatory function, or inflammatory network activity have normalized (19–22). At the cellular level, some endothelial populations may become relatively tolerant, whereas others remain sensitized or readily reactivated. At the systems level, however, the NVU may still persist in a pathological steady state. This is because BBB leakage, endothelial activation by patient-derived plasma, glial amplification loops, oxidative stress, hypoperfusion, and immune-cell recruitment continue to reinforce one another (6, 7, 50, 57, 76, 77, 80, 82–87). Therefore, sensitization/priming and tolerance-like adaptation should be viewed as heterogeneous endothelial response patterns within the broader framework of persistent endothelial dysfunction, not as evidence against the existence of a vicious cycle. This distinction reinforces the central concept of the present review: in CSVD, endothelial dysfunction remains an active driver of ongoing neuroinflammation even if endothelial responses become heterogeneous over time. These considerations also suggest several therapeutic entry points, including endothelial protection, barrier stabilization, and interruption of inflammatory amplification pathways.

### 1.6 Therapeutic strategies

The self-reinforcing interaction between endothelial injury and neuroinflammation represents a rational therapeutic target in CSVD. However, the evidence supporting different candidate interventions is highly uneven. Currently direct randomized human evidence in symptomatic CSVD/lacunar stroke is limited to isosorbide mononitrate/cilostazol and tadalafil. A broader range of licensed drugs—including antihypertensives, statins, antithrombotics, and neuroprotective agents—are being investigated, but most have not yet been tested in CSVD−specific phase II/III trials with clinical or imaging endpoints (94, 95). By contrast, natalizumab and edaravone dexborneol have been tested clinically in acute ischemic stroke rather than chronic CSVD, whereas Phosphoinositide 3-kinase (PI3K) inhibition, salidroside, tetracycline-class/MMP-directed strategies, prostaglandin E receptor 3 (EP3) modulation, mesenchymal stem cells (MSCs), and granulocyte colony-stimulating factor (G-CSF) remain supported largely by animal or other preclinical studies (90–92, 96–116). Accordingly, these therapies should not be discussed as a single class of uniformly “promising” interventions; rather, their interpretation should depend on whether the evidence derives from human CSVD, non-CSVD human disease, or experimental models. This distinction is essential because benefits observed in acute injury paradigms or single animal models cannot be assumed to translate into heterogeneous, chronic human CSVD (3, 94, 95).

#### 1.6.1 Targeting endothelial dysfunction: addressing eNOS uncoupling and oxidative stress

Among endothelial-directed strategies, the most direct human CSVD evidence currently comes from the LACI-2 trial. This trial enrolled 363 patients with symptomatic CSVD/lacunar stroke. It found that isosorbide mononitrate (a nitric oxide donor) and cilostazol (a PDE3 inhibitor with vasodilatory and antiplatelet effects) were feasible, well tolerated, and not associated with major safety concerns. Exploratory analyses suggested possible reductions in recurrent vascular events, dependence, and cognitive impairment (90). However, as a phase II feasibility study, LACI-2 was not powered to detect efficacy definitively, its exploratory efficacy signals require confirmation in adequately powered phase III trials. Therefore, these findings should be interpreted as encouraging but not yet practice-changing.

By contrast, the phosphodiesterase-5 (PDE5) inhibitor tadalafil has produced mixed and overall inconclusive results. In a small crossover study of patients with small-vessel occlusion stroke, a single 20 mg dose increased cortical microvascular oxygen saturation, but no significant improvement was observed in transcranial Doppler parameters or peripheral endothelial markers (91). More importantly, the phase II ETLAS-2 trial found that tadalafil failed to meet the prespecified compliance target. It was associated with lower adherence and more frequent adverse events than placebo, and did not improve cognition, mental well-being, or blood pressure; only a nonsignificant trend toward reduced white matter hyperintensity progression was observed (92). Thus, nitric oxide/cyclic guanosine monophosphate (NO/cGMP)-enhancing approaches currently provide either exploratory positive signals (isosorbide mononitrate/cilostazol) or inconclusive/negative feasibility-efficacy signals (tadalafil), rather than definitive disease-modifying evidence for CSVD.

Preclinical support for targeting upstream oxidative and endothelial signaling remains hypothesis-generating. In aged Col4a1 mutant mice, PI3K blockade restored neurovascular coupling defects and improved memory performance (96). However, this evidence derives from a monogenic mouse model rather than sporadic human CSVD. Likewise, oxidative-stress-directed agents such as edaravone dexborneol have shown beneficial effects in experimental vascular dementia or CSVD-related models (104, 114, 116). But the strongest current clinical evidence for edaravone dexborneol comes from acute ischemic stroke rather than chronic CSVD (106). Therefore, although oxidative and endothelial signaling are mechanistically attractive therapeutic targets, current evidence does not validate these approaches as clinically validated therapies for heterogeneous human CSVD.

#### 1.6.2 Preserving blood-brain barrier integrity: inhibiting disruption and inflammatory leakage

Strategies aimed at preserving blood-brain barrier integrity remain largely preclinical. The most direct human data come from the MINERVA trial, in which minocycline did not reduce BBB permeability or microglial activation in symptomatic CSVD (phase II, negative) (93). In the spontaneously hypertensive rat with two-vessel gradual occlusion (SHR-2VGO) model of CSVD, salidroside attenuated blood-brain barrier disruption and memory impairment and was linked mechanistically to activation of the Notch/ITGB1 pathway (97). Other emerging preclinical strategies include the S1PR1 agonist SEW2871, which reduced BBB leakage and white matter lesions in a rat CSVD model (117), and activation of the KEAP1/NRF2 pathway by tert−butylhydroquinone, which prevented BBB impairment in Trim47−deficient mice (118). However, this evidence is derived from a single rat model using surrogate endpoints rather than human CSVD outcomes.

A similar translational limitation applies to tetracycline-class/MMP-directed strategies. The cited tetracycline study showed suppression of TNF-α/interleukin-6 (IL-6) expression together with inhibition of NF-κB/autophagy signaling in a pretreatment ischemia model (98), whereas the cited doxycycline study showed reduced MMP-9 activity and approximately 25% less blood-brain barrier hyperpermeability in traumatic brain injury (99). These results support biological plausibility, but they do not constitute evidence in chronic CSVD. Moreover, tetracyclines have pleiotropic anti-inflammatory, anti-apoptotic, antioxidant, and metalloproteinase-inhibitory actions (107–111), making it difficult to attribute any apparent benefit specifically to MMP inhibition. Long-term efficacy and safety in human CSVD therefore remain unresolved.

#### 1.6.3 Modulating neuroinflammatory signaling: attenuating amplification cascades

Among anti-inflammatory approaches, the EP3 pathway has CSVD-relevant preclinical support. In stroke-prone renovascular hypertensive rats, EP3 deficiency attenuated small-artery remodeling, reduced extracellular-matrix overexpression via transforming growth factor beta 1 (TGF-β1)/Smad signaling, improved cerebral blood flow, and ameliorated cognitive impairment (100). However, this evidence comes from constitutive receptor deletion rather than from a pharmacological intervention tested in diseased animals or patients. Currently, no direct human evidence supports EP receptor modulation in CSVD (113).

NLRP3 inflammasome inhibition by the selective inhibitor MCC950 in spontaneously hypertensive rats (a chronic CSVD model) suppressed NLRP3 activation, reduced IL-1β and IL-18 production, and attenuated microglial/astrocytic activation. It also improved BBB breakdown, white matter injury, endothelial dysfunction, and cognitive decline (79). These findings position NLRP3 as a mechanistically attractive target, but human evidence remains absent.

In a chronic cerebral hypoperfusion model (bilateral carotid artery stenosis), salidroside promoted microglial polarization from M1 to M2 phenotype, reduced pro-inflammatory cytokines, and attenuated cognitive deficits (119). IL-1β neutralization in a chronic progressive hypoperfusion model largely eliminated grey matter damage and reduced white matter injury without affecting CBF (120). Minocycline reduced microgliosis in chronic hypoperfusion models (121). However, the negative phase II MINERVA trial in symptomatic CSVD patients showed no reduction in BBB permeability or microglial activation (93). This discrepancy highlights the poor translational success of anti-inflammatory strategies from preclinical models to human CSVD.

MSCs likewise remain experimental. In a chronic hypertension-induced rat CSVD model, MSC treatment improved cognitive performance, reduced Aβ deposition, restored aquaporin-4 (AQP4) polarity, and attenuated neuroinflammation, but did not improve white-matter lesions (102). This dissociation suggests partial biological benefit without clear reversal of the structural white-matter substrate. In addition, MSC translation is constrained by source and donor heterogeneity, culture-dependent variability, manufacturing and potency-standardization challenges, biodistribution uncertainty, and unresolved long-term safety issues (103).

Finally, G-CSF and edaravone dexborneol also remain insufficiently validated for CSVD. G-CSF improved blood-brain barrier injury and nonspatial memory in spontaneously hypertensive rats, but no significant change in claudin-5 was observed and no human CSVD data are available (105). Edaravone dexborneol improved neuroinflammatory or fibrotic readouts in experimental vascular dementia/CSVD-related models (104, 114, 116), but no human CSVD data have been reported.

#### 1.6.4 Translational challenges and overall perspective

Most positive findings for CSVD therapies still derive from preclinical models. These models capture only selected disease aspects and rely on surrogate endpoints. By contrast, human CSVD evolves slowly under multiple vascular and genetic factors (3, 94, 95). This mismatch likely explains why many promising experimental results fail to translate into clinical benefit. At present, isosorbide mononitrate/cilostazol provides the most direct and encouraging human signal in symptomatic CSVD, though still exploratory (90). Other strategies, including anti-inflammatory, BBB-protective, and cell-based approaches, remain preclinical or hypothesis-generating (96–105, 107–116). Future progress requires CSVD-specific trials with better phenotyping and clinically meaningful outcome.

These translational constraints also mean that the proposed vicious-cycle framework should distinguish the core evidence-supported mechanism from the more provisional component. **Table 1** summarizes major strategies, their evidence context, and limitations.

**Table 1:** Therapeutic strategies targeting the endothelial dysfunction-neuroinflammation network in CSVD: mechanisms, evidence context, and translational limitations.

Therapeutic target	Representative strategy	Study context	Proposed mechanism	Evidence context	Key translational limitation
Endothelial dysfunction / impaired NO signaling	Isosorbide mononitrate + cilostazol	Symptomatic CSVD / lacunar stroke; LACI-2 phase II trial	NO-donor support plus PDE3-mediated vasodilatory and antiplatelet effects	Direct human CSVD/lacunar-stroke evidence; exploratory positive signal	Feasibility and safety data; not powered for definitive efficacy; phase III confirmation required
Endothelial dysfunction / NO-cGMP pathway	Tadalafil	Small-vessel occlusion stroke pilot; ETLAS-2 phase II CSVD trial	PDE5 inhibition to augment NO/cGMP signaling and microvascular function	Direct human CSVD/lacunar-stroke evidence, but inconclusive or negative	Lower adherence and more adverse events; no clear cognitive, blood-pressure, or clinical benefit
Oxidative stress / endothelial signaling	PI3K inhibition	Aged Col4a1 mutant mouse model	Restore neurovascular coupling and endothelial-related neurovascular function	Preclinical only	Evidence derives from a monogenic model; relevance to sporadic human CSVD remains uncertain
Oxidative stress / neurovascular protection	Edaravone dexborneol	Acute ischemic stroke trials; experimental vascular dementia / CSVD-related models	Reduce oxidative stress and downstream inflammatory or fibrotic injury	Indirect human evidence + preclinical CSVD-related evidence	Human clinical evidence comes from acute ischemic stroke; no direct chronic CSVD validation
BBB protection / neuroinflammatory modulation	Salidroside	Preclinical SHR-2VGO rat CSVD model; chronic cerebral hypoperfusion model	Support Notch/ITGB1-associated BBB repair; promote M1-to-M2 microglial polarization and reduce pro-inflammatory cytokines	Preclinical only	Animal models and surrogate endpoints; no direct human CSVD validation
BBB disruption / S1PR1 pathway	SEW2871 (S1PR1 agonist)	Rat CSVD model	Stabilize endothelial barrier function and reduce BBB leakage and white-matter lesion burden	Preclinical only	Single-model evidence; no clinical validation in human CSVD
BBB disruption / KEAP1-NRF2 oxidative-stress pathway	tert-Butylhydroquinone / KEAP1-NRF2 activation	Trim47-deficient mouse model	Activate KEAP1/NRF2 signaling to protect BBB integrity	Preclinical only	Model-specific mechanistic evidence; applicability to sporadic human CSVD remains unclear
BBB disruption / MMP-associated barrier injury	Tetracycline-class / MMP-directed strategies (e.g., minocycline, doxycycline)	Ischemia, TBI, and chronic hypoperfusion models; MINERVA phase II symptomatic CSVD trial	Suppress inflammatory signaling and MMP activity; reduce BBB injury or microgliosis in models	Preclinical support + direct negative human CSVD evidence	MINERVA showed no reduction in BBB permeability or microglial activation; pleiotropic effects limit MMP-specific interpretation
Neuroinflammatory amplification / leukocyte recruitment	Natalizumab	Acute ischemic stroke trial	Block alpha4-integrin-dependent immune-cell infiltration	Indirect human evidence	Tested in acute ischemic stroke rather than chronic CSVD; no direct CSVD efficacy validation
Neuroinflammatory amplification / prostaglandin signaling	EP3 modulation	Stroke-prone renovascular hypertensive rat model; receptor-deficiency paradigm	Disrupt PGE2-EP3 signaling and TGF-beta1/Smad-related vascular remodeling	Preclinical only	Mainly receptor-deficiency animal data; no pharmacological or patient-level CSVD validation
Neuroinflammatory amplification / NLRP3 inflammasome	MCC950	Spontaneously hypertensive rat chronic CSVD model	Inhibit NLRP3 activation; reduce IL-1beta/IL-18, glial activation, BBB injury, white-matter injury, endothelial dysfunction, and cognitive decline	Preclinical only	Mechanistically attractive, but human CSVD efficacy and safety remain untested
Neuroinflammatory amplification / IL-1beta signaling	IL-1beta neutralization	Chronic progressive hypoperfusion model	Reduce cytokine-driven tissue injury and attenuate grey- and white-matter damage	Preclinical only	Did not correct underlying CBF reduction; no human CSVD validation
Neuroinflammatory amplification / immune microenvironment	Mesenchymal stem cells (MSCs)	Chronic hypertension-induced rat CSVD model	Attenuate neuroinflammation, reduce Abeta deposition, and restore AQP4 polarity	Preclinical only	Biological and cognitive benefits without clear reversal of white-matter lesions; manufacturing, potency, biodistribution, and long-term safety issues remain
Endogenous repair / neurovascular protection	G-CSF	Spontaneously hypertensive rat model	Support BBB protection and endogenous repair responses	Preclinical only	Animal benefit not validated in human CSVD; no significant claudin-5 change

Abeta, amyloid-beta; AQP4, aquaporin-4; BBB, blood-brain barrier; CBF, cerebral blood flow; cGMP, cyclic guanosine monophosphate; CSVD, cerebral small vessel disease; EP3, prostaglandin E receptor 3; G-CSF, granulocyte colony-stimulating factor; IL, interleukin; ITGB1, integrin subunit beta 1; KEAP1, Kelch-like ECH-associated protein 1; MMP, matrix metalloproteinase; MSCs, mesenchymal stem cells; NLRP3, NLR family pyrin domain containing 3; NO, nitric oxide; NRF2, nuclear factor erythroid 2-related factor 2; PDE, phosphodiesterase; PGE2, prostaglandin E2; PI3K, phosphoinositide 3-kinase; S1PR1, sphingosine-1-phosphate receptor 1; SHR-2VGO, spontaneously hypertensive rat with two-vessel gradual occlusion; TBI, traumatic brain injury; TGF-beta1, transforming growth factor beta 1.

Evidence contexts distinguish direct human CSVD/lacunar-stroke studies from indirect human studies in related conditions and preclinical models. The table summarizes major strategies discussed in the Therapeutic Strategies section, not all drugs under investigation.

## 2 Discussion and outlook

Current evidence supports a recurrent pathogenic architecture in which vascular risk exposures disrupt endothelial homeostasis, promote endothelial phenotypic switching, impair the BBB, and amplify inflammatory signaling within the NVU (4–7, 19–22, 57, 66, 78–81). This sequence is unlikely to apply identically to every CSVD subtype, but it provides a useful framework for organizing findings that otherwise remain fragmented across patient cohorts and experimental models. Several points remain unresolved. Endothelial programs vary markedly along the arteriovenous axis, and capillary BBB-specialized states may be particularly vulnerable to ageing, abnormal flow, and pericyte loss; however, it remains uncertain whether this fully explains the segmental distribution of human CSVD (11–18). Repeated inflammatory stimulation can also induce memory-like endothelial reprogramming, with later responses shifting toward sensitization/priming or tolerance-like adaptation, but comparable heterogeneous endothelial states have not yet been demonstrated directly in human CSVD tissue (19–22).

Even so, current human data support persistence at the systems level: BBB leakage predicts cognitive decline, cerebrovascular dysfunction predicts imaging progression, BBB permeability and microglial activation may coexist without complete spatial overlap, and plasma from patients with early CSVD can activate inflammatory pathways in brain endothelial cells (82–85). These observations support the core model while also defining its present limits. They also have practical implications for biomarker interpretation and therapy. Interventions aimed at endothelial function, BBB integrity, or inflammatory amplification are biologically plausible, but most available evidence remains preclinical or derives from adjacent clinical contexts rather than CSVD itself (90–92, 97–116). Progress will therefore depend on biomarker-guided and subtype-aware studies, stronger temporal resolution, and spatially resolved analyses of human small-vessel lesions (8, 33, 37, 122–133).
